# Pathways for reduction of HIV‐related stigma: a model derived from longitudinal qualitative research in Kenya and Uganda

**DOI:** 10.1002/jia2.25647

**Published:** 2020-12-07

**Authors:** Carol S Camlin, Edwin D Charlebois, Monica Getahun, Cecilia Akatukwasa, Frederick Atwine, Harriet Itiakorit, Robert Bakanoma, Irene Maeri, Lawrence Owino, Anjeline Onyango, Gabriel Chamie, Tamara D Clark, Craig R Cohen, Dalsone Kwarisiima, Jane Kabami, Norton Sang, Moses R Kamya, Elizabeth A Bukusi, Maya L Petersen, Diane V Havlir

**Affiliations:** ^1^ Department of Obstetrics Gynecology & Reproductive Sciences University of California San Francisco San Francisco CA USA; ^2^ Department of Medicine Center for AIDS Prevention Studies University of California San Francisco San Francisco CA USA; ^3^ Infectious Diseases Research Collaboration Kampala Uganda; ^4^ Kenya Medical Research Institute Nairobi Kenya; ^5^ Division of HIV Department of Medicine Infectious Diseases, and Global Medicine University of California San Francisco San Francisco CA USA; ^6^ School of Medicine Makerere University Kampala Uganda; ^7^ Divisions of Biostatistics and Epidemiology School of Public Health University of California Berkeley Berkeley CA USA

**Keywords:** HIV‐related stigma, HIV testing, HIV treatment, Universal Testing and Treatment, sub‐Saharan Africa, community

## Abstract

**Introduction:**

The rollout of antiretroviral therapy (ART) has been associated with reductions in HIV‐related stigma, but pathways through which this reduction occurs are poorly understood. In the newer context of universal test and treat (UTT) interventions, where rapid diffusion of ART uptake takes place, there is an opportunity to understand the processes through which HIV‐related stigma can decline, and how UTT strategies may precipitate more rapid and widespread changes in stigma. This qualitative study sought to evaluate how a UTT intervention influenced changes in beliefs, attitudes and behaviours related to HIV.

**Methods:**

Longitudinal qualitative in‐depth semi‐structured interview data were collected within a community‐cluster randomized UTT trial, the Sustainable East Africa Research in Community Health (SEARCH) study, annually over three rounds (2014 to 2016) from two cohorts of adults (n = 32 community leaders, and n = 112 community members) in eight rural communities in Uganda and Kenya. Data were inductively analysed to develop new theory for understanding the pathways of stigma decline.

**Results:**

We present an emergent theoretical model of pathways through which HIV‐related stigma may decline: *internalized stigma* may be reduced by two processes accelerated through the uptake and successful usage of ART: first, a reduced fear of dying and increased optimism for prolonged and healthy years of life; second, a restoration of perceived social value and fulfilment of subjective role expectations via restored physical strength and productivity. *Anticipated stigma* may be reduced in response to widespread engagement in HIV testing, leading to an increasing number of HIV status disclosures in a community, “normalizing” disclosure and reducing fears. Improvements in the perceived quality of HIV care lead to people living with HIV (PLHIV) seeking care in nearby facilities, seeing other known community members living with HIV, reducing isolation and facilitating opportunities for social support and “solidarity.” Finally, *enacted stigma* may be reduced in response to the community viewing the healthy bodies of PLHIV successfully engaged in treatment, which lessens the fears that trigger enacted stigma; it becomes no longer socially normative to stigmatize PLHIV. This process may be reinforced through public health messaging and anti‐discrimination laws.

**Conclusions:**

Declines in HIV‐related stigma appear to underway and explained by social processes accelerated by UTT efforts. Widespread implementation of UTT shows promise for reducing multiple dimensions of stigma, which is critical for improving health outcomes among PLHIV.

## 1 Introduction

The scale‐up of antiretroviral therapy (ART) is significantly associated with reductions in HIV‐related stigma: there is evidence from Demographic and Health and AIDS Indicator Survey data that HIV‐related stigma has declined, especially in sub‐Saharan African countries with high HIV prevalence and ART uptake [1, 2]. HIV stigma appears to have decreased since the introduction of universal ART in Botswana [3]; ARV availability and uptake were found to reduce internalized stigma among people living with HIV (PLHIV) in rural Tanzania [4] and Uganda [5]. There is, however, counter‐evidence that HIV stigma persists in many settings, even in contexts of the ART rollout [6, 7]; and, results from a five‐country study were equivocal, suggesting that although increased access to ART may lower HIV stigma, stigma was found to have declined in only two of the five sites in which ART was scaled‐up [8]. A recent review of research on HIV stigma and HIV testing in the 90‐90‐90 era highlighted the role of persistent stigma in shortfalls towards achieving testing targets (the “first 90”) across sub‐Saharan Africa [9].

Expanding upon Goffman’s original conceptualization of stigma as a social process through which a person’s identity is rendered “spoiled” [10] there is a rich theoretical literature on the dimensions of stigma [11], measurement of HIV‐related stigma [12, 13, 14, 15], the social processes and structural inequalities (including stigmatizing community norms and practices, institutional policies and laws) that produce and reinforce HIV‐related stigma [16, 17], and on the pathways through which stigma leads to adverse health outcomes for PLHIV [18, 19]. However, prior theoretical work on the social and behavioural pathways through which stigma may be reduced is limited and is primarily found in the literature on stigma reduction interventions. The theoretical basis of many stigma interventions has not been well‐described or has been imprecise vis‐à‐vis how the interventions intend to shift aspects of stigma that are harmful to health [18], with exceptions: a community‐based intervention in Zimbabwe designed to reduce internalized stigma found that experiences with the intervention facilitated disclosure of HIV status, reduced feelings of life limitations due to HIV, and a greater positive mentality among PLHIV [20]. Two scoping reviews of stigma interventions have been published that focused on categorizing programmatic approaches, with less attention to mechanisms of stigma reduction: Brown and colleagues reviewed 22 stigma intervention studies and identified information‐based approaches, skills‐building, counselling and contact with other PLHIV [21]. Thaka and colleagues reviewed 34 stigma intervention studies that were designed to influence HIV testing uptake, and categorized intervention strategies into four types: awareness creation, influencing normative behaviour, providing support and developing regulatory laws [22]. They proposed that testing uptake was achieved via two broad pathways: improved knowledge of HIV leading to changes in stigmatizing attitudes, and improved knowledge and attitudes leading to changes in stigmatizing behaviours [23].

There has been less attention paid to understanding the processes through which HIV‐related stigma can decline, and in the newer, larger context of universal test and treat (UTT) interventions, where rapid diffusion of HIV status and ART uptake takes place, there is an important opportunity to understand the processes through which HIV‐related stigma can decline and how UTT strategies may precipitate more rapid and widespread changes in stigma.

This study proposes an explanatory model of pathways through which multiple dimensions of HIV‐related stigma may be reduced in the context of large‐scale treatment rollout. We draw upon an interpretivist theoretical approach [24] to propose a new theory of pathways of stigma reduction, developed on the basis of empirical findings from a qualitative study conducted in Kenya and Uganda from 2014 through 2016. The study was embedded within a community‐cluster randomized UTT trial, the Sustainable East Africa Research in Community Health (SEARCH) study (NCT01864603) in 32 communities across three regions of Uganda and Kenya. SEARCH demonstrated that its interventions of hybrid mobile testing campaigns [25] and “streamlined care” led to testing, linkage and viral suppression rates that surpassed the 90‐90‐90 goals after three years [26, 27]. This study sought, overall, to evaluate how UTT influenced changes in beliefs, attitudes and behaviours related to HIV.

## 2 Methods

**Figure 1:**
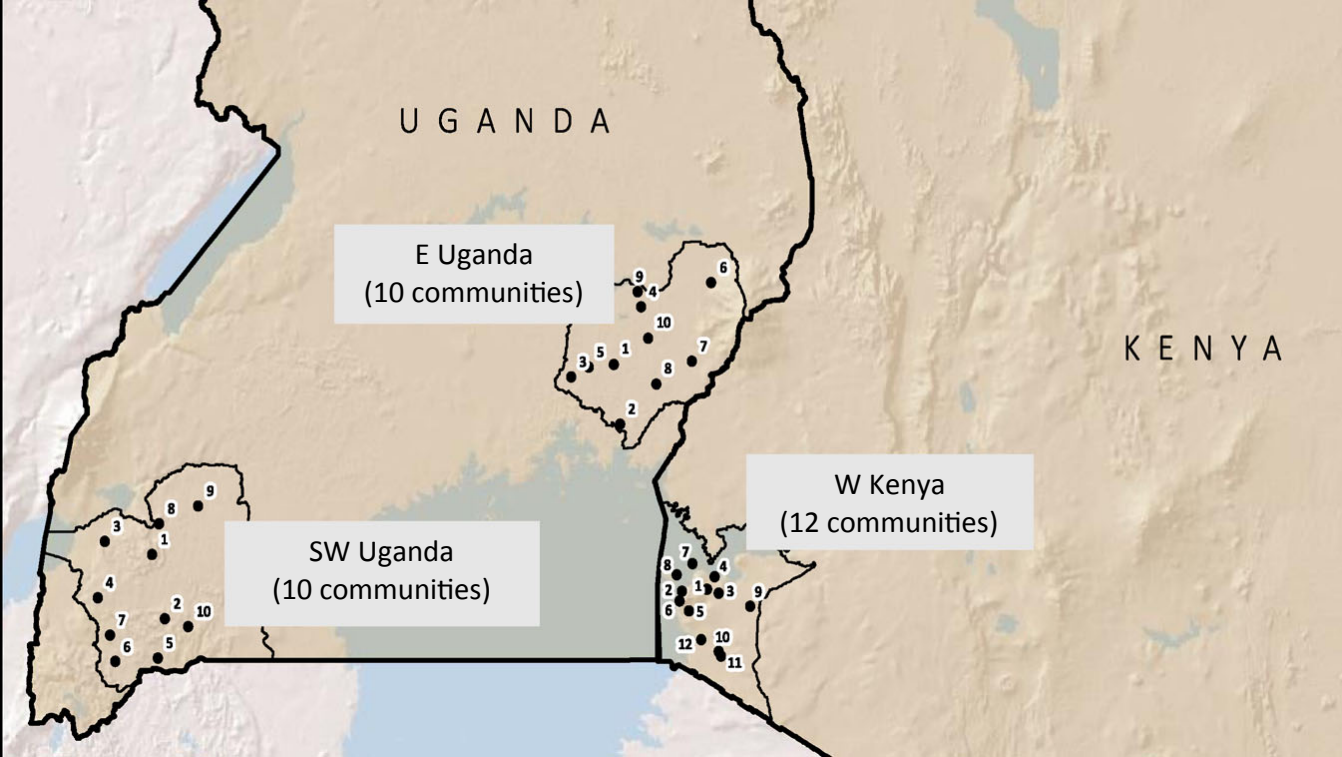
Map of communities participating in the Sustainable East Africa Research in Community Health (SEARCH) study.

For this analysis, we used longitudinal qualitative data to examine the evidence for the change in dimensions of HIV‐related stigma, and the pathways through which any changes may have occurred over time, in SEARCH communities (Figure 1). This analysis used qualitative in‐depth semi‐structured interview data collected annually over three rounds (2014 to 2016) from two cohorts of adults (community members and leaders) living in 8 of the 32 communities across the three regions (4 intervention and 4 control arm communities).

### 2.1 Sampling methods

Cohorts were composed using purposive and systematic sampling methods: n = 32 community leaders were selected purposively for gender balance from among the local leaders engaged by SEARCH to help mobilize communities (selected for the cohort independent of their HIV status), and n = 112 community members were systematically selected (systematic progression through a list of eligible participants organized by strata) from a stratified random sub‐sample of HIV‐positive and ‐negative community members. Table 1 shows the numbers recruited and retained in the two cohorts across data collection rounds.

**Table 1:** Qualitative cohorts and data collection rounds, Sustainable East Africa Research in Community Health (SEARCH) study

Cohort	Baseline year (February to November 2014)				Year 2^a^ (February 2015 to Jan. 2016)				Year 3^b^ (February to November 2016)			
	n = 112				n = 108				n = 103			
Community members^c^	Male 47	Female 65	HIV + 70	HIV − 42	Male 45	Female 63	HIV + 65	HIV − 43	Male 44	Female 59	HIV + 63	HIV − 40
Community leaders^d^	n = 32				n = 32				n = 32			
	Male 20		Female 12		Male 19		Female 13		Male 19		Female 13	

^a^ Year 2 notes: n = 4 not included due to death, out‐migration, loss to follow‐up (LTFU) or refusal; 1 HIV‐negative individual replaced; 1 community leader replaced

^b^ year 3 notes: n = 5 not included after year 1 due to death, out‐migration, LTFU or refusal; no replacements

^c^ randomly selected community members stratified by HIV status (5 HIV negative and 9 PLHIV per community)

^d^ community leader HIV status not known to qualitative team at the time of data collection; selected based on role in the community and involvement in Sustainable East Africa Research in Community Health (SEARCH) study mobilization.

### 2.2 Data collection

Data were collected at baseline (February to November 2014), one‐year follow‐up (February 2015 to January 2016) and two‐year follow‐up (February to November 2016), by a gender‐balanced team of trained qualitative researchers who were native speakers of local languages, using in‐depth semi‐structured interview (IDI) guides. Written informed consent was obtained from all study participants. Interview guides explored dimensions of HIV stigma via explorations of individual attitudes, beliefs and experiences, including explorations of perceived community norms, conversations about HIV, disclosure expectations and experiences, care engagement processes, and perceptions of SEARCH intervention elements. Follow‐up year IDI guides were tailored with individualized follow‐up questions based on the previous year responses. (Interview guides and summary sheets are included as Supplementary Materials.) The local team members also transcribed and translated audio recordings.

### 2.3 Analysis methods

We used a systematic interpretivist approach, in the domain of theory‐generative research [24], which involves both “abductive” and “inductive” analyses of empirical data and production of findings, to foster theory construction and generate new explanatory hypotheses. The approach is akin to constructivist grounded theory [28] in that it involves iterative rounds of coding of textual data, but does not foreground a pre‐existing heuristic or model; rather, it requires in‐depth familiarity with a range of sociological and behavioural theories. We particularly drew upon theories of stigma as a broad framework upon which to build [10, 16]. The lead investigator, project coordinator and team members collaboratively defined an analytical coding framework following review and discussion of the first sets of transcripts (Table S1). Using Atlas.ti software [29], the team coded the full set of n = 419 transcripts, and iteratively refined the coding framework at defined periods during data collection. Codes were extracted as query reports, which were used to review and reduce data into coherent analytical categories. An analysis matrix was developed to summarize themes emergent in the data, along with illustrative quotes categorized by data source. A final inductive analysis was undertaken by the lead investigator and co‐investigator with n = 107 transcripts, selected for the quality of information shared, thematic richness and depth. Case‐level (i.e. within‐individual) coding was applied to categorize the quality of evidence of the direction of changes in dimensions of stigma (whether decreased, increased, or no apparent change), and to characterize pathways of stigma reduction, with attention to contradictory evidence and deviant cases.

### 2.4 Ethical approvals

This research received ethical approval from the University of California San Francisco Committee on Human Research, the Ethical Review Committee of the Kenya Medical Research Institute, the Makerere University School of Medicine Research and Ethics Committee and the Uganda National Council for Science and Technology.

## 3 Results

**Figure 2:**
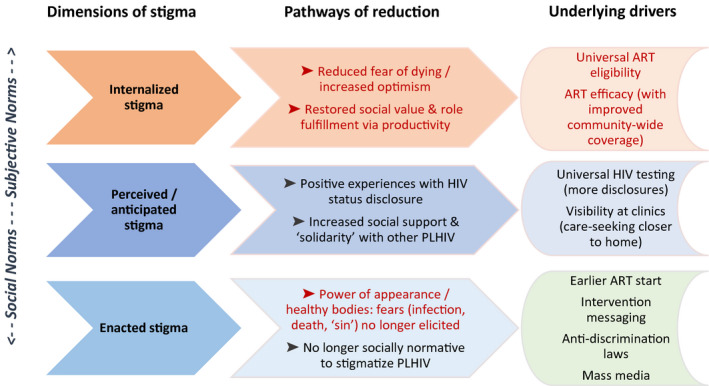
The Pathways Model for HIV‐Related Stigma Reduction. Text in red denotes thematic linkage: Wider uptake and successful usage of ART acted to reduce forms of both internalized and enacted stigma via multiple pathways. ART, Antiretroviral therapy; PLHIV, People living with HIV

Evidence for reductions in multiple types of HIV stigma, both internalized and within communities, was noted in participants’ accounts, with participants describing changes they had observed over time. It was also derived from our own perceptions of divergences over time, from our within‐case analysis of content of the narratives. This evidence informed our proposed theoretical model of pathways for stigma reduction during large‐scale UTT implementation (Figure 2).

### 3.1 The pathways model for HIV‐related stigma reduction

We hypothesize that internalized stigma, or “self‐stigma” (where negative self‐appraisals among PLHIV are endorsed and lead to feelings of shame, self‐blame and low self‐worth), may be reduced in response to two main processes, both of which may be accelerated through the uptake and successful usage of ART (including, especially in the context of UTT, earlier usage): first, a reduced fear of dying and increased optimism for prolonged and healthy years of life, which relieves the sadness and fearful resignation previously associated with having HIV. An element of vicarious efficacy may contribute to this pathway, in that uptake and successful usage of ART by others in the community may contribute to optimism and positive expectancies for ART usage. Second, internalized stigma may be reduced via a restoration of one’s perceived social value and fulfilment of subjective role expectations (e.g. as spouses, parents, household providers, workers, leaders), through the direct experience of restored physical strength and productivity as a result of successful treatment engagement.

We hypothesize that anticipated stigma (where individuals expect that others will treat them poorly because of an HIV‐positive status) may be reduced in response to two main processes: first, widespread engagement in HIV testing may lead to an increasing number of HIV status disclosures in a community, in effect “normalizing” disclosure; this may then contribute to a greater number of positive experiences with disclosure among community members. Narratives shared among community members about positive disclosure experiences may reduce fears. (In our narratives, there were counter‐examples, especially among women.) Second, as treatment services are made more patient‐centred and flexible, seeking HIV care closer to home may be made more attractive than it was in the past (when PLHIV, particularly men, purposefully sought care away from home communities to avoid disclosure); as a result, PLHIV see other known community members living with HIV at the same local clinic, which may reduce social isolation and increase opportunities for social support and “solidarity” with other PLHIV, which in turn may act to reduce anticipated stigma and disclosure fears.

Finally, we hypothesize that enacted stigma (experiences of discrimination, devaluation, social exclusion or violence as a result of HIV‐positive status) may be reduced in response to the visible, tangible power of appearance of healthy bodies of PLHIV who are successfully engaged in treatment: as efficacious ART visibly improves health, the fears that trigger enacted stigma (of infection and death, or associations of physical decay with moral decay or “sin,” in the context of HIV) may be less often elicited. In tandem, it may become no longer socially normative to stigmatize PLHIV. Normative change in socially acceptable speech about PLHIV may be further reinforced through intervention messaging (whether delivered by community leaders, community health workers, HIV clinic staff or study staff), by mass media campaigns that encourage support for PLHIV along with testing and treatment, and with anti‐discrimination laws.

Excerpts from interview narratives are presented here to illustrate the pathways through which forms of HIV‐related stigma may have declined.

### 3.2 Pathways of reductions in internalized stigma

The first set of narratives support *a reduced fear of dying and increased optimism* and positive expectations for the future as a result of widespread ART uptake. A Kenyan woman living with HIV (age 36) shared:

*Baseline:* […] It is said that those on HIV care die sudden death.

*Year 2:* People’s feelings have changed. […] I still have a heavy heart to start HIV care, but […] somebody is seeing so‐and‐so who had deteriorated health‐wise looking very healthy, will say, “I can also start on ARVs treatment and look like so‐and‐so, or even look better than her.” [*Interviewer: Why do you think these changes have occurred*?] […] It is the availability of these drugs that has made these changes occur.”

A Kenyan man living with HIV (age 38) described growing interest in ART as a result of having seen many other PLHIV restored to health. He described how PLHIV are helping others to start ART:

*Year 2:* People’s feelings towards HIV have changed this way, sometimes when you are seeing somebody who is looking very sick, you will create time to go and talk to this person and convince him to go for HIV test, and if he is found HIV positive, to start on care. This is something that people are doing, trying to encourage people to go for testing and start on care in order for them to survive longer […] If I had not been taking ARVs then I would not be in this world.

Along with discussions reflecting a reduced fear of dying, community members discussed accumulated experience of seeing others die as a result of discontinuing care. A Kenyan woman (age 31) discussed this negative reinforcement of HIV care engagement:

*Year 2*: These days people are not scared of taking drugs, because they have seen death among those who have dropped care. When people attend funerals, they hear people talk about it… that the person being buried had dropped care […] and when you are traveling you carry [ART] with you without any worries. People just carry it openly from the hospital, quite unlike before, when people used to wrap it and hide it from the views of others.

PLHIV also described their own feelings of *restored social value and role fulfilment via their productivity* and strength due to improved health. One Kenyan woman (age 36) said, “ARVs have helped us a lot, it has given us some strength, [so] that if we go to the farm we are more active than even the non‐infected”. Similarly, a Kenyan man (age 28) describes stigma having reduced as a result of restored productivity:

*Baseline:* People who talk ill or gossip are still there, and we cannot avoid it.

*Year 2:* When you are actively involved in your work and carrying on with your duties without fail, [people] will have nothing to talk about you. It’s only bad when you fall sick […] that is when people start talking ill about you. […] I am saying that stigma is less experienced not only in this area, but the whole of Luo Nyanza. [*Region in Kenya*].

This is reinforced by *others’ assessments of improved productivity and the social value of PLHIV* due to widespread successful ART usage. In the following excerpts, a leader of a community in southwestern Uganda initially voiced stigmatizing attitudes towards PLHIV. Over time, her discussions about PLHIV centred less on promiscuity and blame, and more on PLHIV’s productivity and ability to maintain their households. She had also, perhaps, become more open about a family member living with HIV, and had internalized messages that PLHIV should not be openly stigmatized.

*Baseline:* [HIV] is a disease that came out of sin. […] that is what causes someone to hide their status from their relatives, from their husbands or from their wives, because it came from the sin of promiscuity. If it is another disease like hypertension and diabetes you cannot hide it, because you know that is a disease that just comes by itself […] for HIV, people hide it is because you have brought it yourself.

*Year 2:* Some people acquire HIV out of being promiscuous, but there are those that were born with HIV […] so you cannot say that HIV is only for the promiscuous. […] There is a rich man that I know who lost his wife 20 years ago, but now we see his home is stable; his children are going to school. So sometimes when we are talking to people, we use that man as an example saying,“[so‐and‐so] has had HIV for a very long time, isn’t he surviving, his children are surviving and he has made developments in his home?”

Year 3: [Interviewer: Last time, you mentioned that people don’t stigmatize people with HIV because they know that a positive person can live for long, but there are those that look at them as hopeless individuals. Is that kind of thinking still there?] That thinking is no longer there. For example, my brother’s son was born with HIV, but he has lived for 25 years now. He went to school and is now an employed man. So, it is for such reasons that people no longer see HIV‐positive people as hopeless beings. They take it as a disease like any other and it does not affect someone’s productivity.

### 3.3 Pathways of reductions in anticipated stigma

*Positive and more frequent experiences with HIV status disclosure* appeared to prevent or reduce the impact of anticipated stigma. Following community‐wide HIV testing introduced by SEARCH, the content of conversations about HIV among community members changed. During the baseline year, discussions centred on attitudes towards PLHIV. While many individuals reported that attitudes had improved greatly since the early days of the epidemic, others reported persistent anticipated stigma and fears of disclosure. In year two, narratives were composed more of discussions surrounding decision‐making and experiences related to HIV testing and accessing treatment, with a greater apparent level of openness. (See also Table S2.)

A Ugandan woman living with HIV (age 34) in the baseline year described having no discussions with others about HIV and knowing no other PLHIV in her community, and one year later, described having many, frequent discussions and having met twelve other PLHIV:

*Baseline:* Some people fear HIV/AIDS to the extent that they go as far as [distant communities] for the tests or HIV care. When they return, they keep very silent! This is all because of fear of being known as HIV positive. [*Interviewer: Do you know anybody who is HIV positive or who has died of HIV/AIDS?*] Here in [this] parish I am not able to know them because they are still in hiding and have not openly come out […] Some say […] “why don’t you die, rather than people standing a risk of contracting HIV infection from you?”

*Year 2:* [*Interviewer: Last year you also said that people in the village talk badly about PLHIV…*] Nowadays there are few that talk like that. […] they tell them to take ARVs so that they can stay for long and keep their children. […] They don’t see it as a very bad disease— they say that at least HIV/AIDS is better than other diseases […] you can live with it for a longer period of time.

Interviews with a Kenyan community leader (age 30) who is also a Community Health Worker showed changes in his perceptions of anticipated stigma, as more PLHIV were diagnosed and disclosed their status to others:

*Baseline:* In this community […] people are saying, “so‐and‐so is going to see me [at the local health facility]— they are going to say I am like this and this”.

*Year 2:* Initially there used to be discrimination and people could gossip about those who are infected, “so‐and‐so has started using ARVs”. Such talks were very common. I would say that SEARCH has created openmindedness in this community […] You may find that one had talked ill about those living HIV positive […] she or he also turns out to be HIV positive too; this is when they get to appreciate the importance of ARVs. […] It is SEARCH who discovered about everybody’s status and equality and acceptance has really prevailed.

*Year 3:* PLHIV are very healthy and live normal lives, they can go farming and they are able to all the domestic activities with lots of power. […] many community members go for HIV testing without being reminded, seek medical attention on their own in hospitals without being pushed or reminded. […] The fear also subsided. People have seen that those who have been tested HIV positive do not lie in their beds sick.

Perceived or anticipated stigma may also be reduced through *increased social support and “solidarity” with other PLHIV* as greater numbers of PLHIV disclosed their status and sought care closer to home, at facilities where they encountered neighbours. As more individuals experienced the benefits of ART, they advocated for others to initiate ART:

These days the infected approach the newly infected who are still in hiding, and they give them support […] “just be free with your status because the drugs work very well with those who have accepted their status and feel very free to talk about themselves to others”. Male community leader, age 38, Kenya

The drugs [ART] have helped many people… even me who is now talking to you, I would have died long time ago.” HIV+ woman, age 76, Kenya

Community members perceived that SEARCH brought quality HIV care closer to their homes: “in the past, care was expensive because many hospitals were not providing the service and people could travel long distances to go for care. Many could lack transport hence many deaths but now HIV care has been brought closer to the people.” Once PLHIV began to access local facilities, in order to forego the expense of traveling far to access care, meant that others in the community could see them, precipitating more disclosures: “seeing friends at the local facility, that promotes acceptance.” A Kenyan woman (age 41) described how, as anticipated stigma declined, the trade‐off between accessing far facilities to avoid disclosure no longer seemed as advantageous:

*Year 2*: [*Interviewer: What do you think has promoted less fear in accessing HIV care?*] [ARVs] were not available locally and people used to access them in the hard way, they then realized that they were using a lot of money to reach those far facilities. This could even subject some to missing their appointments due to lack of fare. And so, when most of these services were decentralized, most people saw the sense in accessing them locally and no longer hide from others. In these local facilities, people meet familiar friends and this is what also promotes acceptance.

*Year 3*: They [community members] still feel that HIV is not harmful because there are drugs. Someone may have lived on ARVs for even 20 years. [*Interviewer: Since we last spoke have you known any other people in your community who are infected with HIV?*] Yes I know of about three people who I have met at the hospital when I went for my drugs. They were not on [ART] before.

### 3.4 Pathways for reductions in enacted stigma

A separate pathway acting upon enacted stigma (words and actions that are stigmatizing) is the *power of appearance/ healthy bodies* countering fears that drive enacted stigma (of infection, death or “sin”) that are less often elicited or triggered. The positive effects of ART appeared to have emboldened many PLHIV to openly engage in care despite anticipated and enacted stigma:

*Baseline:* People fear [HIV]. Some people do not even admit that they are HIV positive. [*Interviewer: Why do you think people fear HIV/AIDS?*] It is because some community people like to laugh at us […] They say, “that person is about to die!” […] [*Interviewer: Do they say those words when you are hearing?*] Yes, but what I know is that even those people who say those words will die. […] Another reason people fear HIV/AIDS is that they still want to have sex. So when a person shows up to openly disclose his or her HIV status, then partners will refuse them.

*Year 2*: We have some neighbours here they used to abuse us, that AIDS has confused us, that we are sick and about to die, things of that kind. […] This world is round— they were also tested HIV positive, and they have completely kept quiet. [HIV‐positive people] don’t fear, we stay together and even share certain things. […] You see, me, when I started [ART], I was fearing, but now I am free… You only need to adhere to taking your medicine, and you can stay longer, like the years that I have finished now.

*Year 3*: [*Interviewer: How do people in the community look at HIV/AIDS?*] They look at it as a normal disease, and they see it as not to be complicated like it used to be. […] They see HIV‐positive people looking healthy and energetic like them. HIV+ woman, age 54, E Uganda

In some instances, it seems that the healthy bodies of PLHIV successfully engaged in care have changed attitudes. In others, there were signs that over time, it became *no longer socially normative to openly stigmatize PLHIV*. The following narratives among HIV‐negative individuals reveal these changes.

*Baseline:* I think it is brought about by prostitution and promiscuity, do you think that if you are not disorganized you get that illness? It is a disease that you bring upon yourself; it is not like measles that you just get.

*Year 3:* They no longer treat them badly […] because they came to know that the disease is for everyone, and you are not supposed to mistreat another person. How do you harass another person when you know that you are also like that? It’s like getting measles. HIV− woman, age 41, SW Uganda

*Year 3:* Discrimination has also gone down because the Government has imposed law on the discrimination. I have a relative who said to the other, “I will kill you before that HIV of yours kills you”. He was reported to the authorities and charged accordingly. This has created fear in many people and they don’t just anyhow abuse the infected ones. This is also because the HIV positives look healthier than the negatives because of the ARVs. HIV− woman, age 49, Kenya

### 3.5 Counter‐evidence

We observed consistency across countries and regions in the suggested pathways for HIV associated stigma. However, there were counter‐examples, showing that anticipated stigma and perhaps stigmatizing attitudes persisted in some cases. An HIV‐ Kenyan woman (age 34) said, “Yes there are people who, maybe their [HIV‐positive status] hadn’t been revealed [before]. So if theirs have been revealed this year, now they want to hide. They don’t want to take drugs where others are taking, they want to use a motorbike to go and take it from Homa Bay or even Sori so that […] the file may not be here for these people to know.” The following excerpts from interviews with a WLHIV (age 32) in Kenya are illustrative of the few cases in which PLHIV perceived little to no change in stigma over a three‐year period:

*Baseline*: Those suffering from HIV/AIDS are so secretive with their status. They would not like people to know them. […] they see HIV/AIDS as a very bad disease. I can’t tell any other person about my status or even about ARVs… you know once you have told any other person he/she will now walk around with your name spreading it to others.

*Year 2*: People are just taking ARVs but it is something that is done discreetly. Nobody would want to be associated with ARVs. People are not free with taking drugs.

Those who did not feel safe to disclose were relieved that taking ART permitted them to more effectively conceal their status. They described how the appearance of a healthy body gave them “cover,” enabling them to hide their HIV status from others. However, there were reports of enacted stigma against some HIV‐positive individuals following status disclosure, particularly women. Moreover, perceptions that stigma had reduced elicited new anxieties:

*Year 2*: Now you cannot identify the infected… now there is less fear for HIV, and that causes us to worry. [*Interviewer: Why do you worry?*] Because more people are going to get infected… people no longer fear HIV like they used to. Male community leader, SW Uganda

## 4 Discussion

We have presented here a theoretical model of pathways through which dimensions of stigma may have declined in communities, in the context of a large universal testing and treatment trial that brought about a rapid scale‐up of HIV testing, diagnosis, and treatment engagement. Just as we have come to understand that unique dimensions of stigma have differing pathways to and effects on health outcomes for individual PLHIV, with individual‐level dimensions of HIV‐related stigma operating through interpersonal factors, mental health, psychological resources and biological stress pathways [18], the empirical findings suggest that there are be differing pathways operating at individual, interpersonal and community levels that may lead to declines in dimensions of HIV‐related stigma.

In summary, *internalized stigma* may be reduced in response to two processes set into motion through the uptake and successful usage of ART, (including earlier usage as a result of broadened ART eligibility): first, a reduced fear of dying and increased optimism for prolonged and healthy years of life (vicarious efficacy may contribute to this pathway through observations of successful usage of ART by others in the community); second, a restoration of perceived social value and fulfilment of subjective role expectations (e.g. as household providers), via restored physical strength and productivity. *Anticipated stigma* may be reduced in response to two processes: first, widespread engagement in universal HIV testing leads to an increasing number of HIV status disclosures in a community, “normalizing” being HIV positive and disclosing this, and reducing fears. Secondly, improvements in the perceived quality of HIV care may lead to PLHIV seeking care in nearby facilities rather than away from home to avoid disclosure; as a result, seeing other known community members living with HIV may reduce isolation and increasing opportunities for social support and “solidarity.” Finally, *enacted stigma* may be reduced in response to viewing the healthy bodies of PLHIV successfully engaged in treatment: fears that trigger enacted stigma (of infection and death, or associations of physical decay with moral decay or “sin”) may be less often elicited, and it may become no longer socially normative to stigmatize PLHIV. This pathway may be reinforced through public health messaging and anti‐discrimination laws.

The potential for UTT interventions to affect stigma has been recognized; one such trial, PopART, included a study designed to assess how stigma affects, and is affected by, implementation of a UTT intervention (with results not yet published) [30]. This study is unique in the literature to date in its intent to comprehensively characterize pathways of stigma reduction in the context of UTT, but elements of the model, and the empirical findings supporting it, confirm prior research. The findings here support our prior observations that growing numbers of PLHIV were seen to be playing an unforeseen and very active role in leading others in their communities into testing and treatment— and, we proposed, this phenomenon was driven by its power to permit PLHIV to shed stigmatized social identities, restore their social value and regain full personhood in their communities [31]. Related to this, our findings support prior research suggesting that interventions that are designed to facilitate social support among PLHIV may act powerfully to help PLHIV overcome internalized stigma: studies in Tanzania and South Africa suggested that increased ART access led to a “normalization” of the disease, often a result of seeing others regain health and resume economic activities, and of receiving social and emotional support from health providers and other PLHIV [32, 33, 34, 35].

This study also confirms prior research showing that renewed physical vigour as a result of ART itself leads to restored productivity among PLHIV, which in turn restores social status and perceived role fulfilment (which would act upon multiple dimensions of stigma). Thus, unsurprisingly, a livelihood intervention designed to improve health outcomes among PLHIV in Kenya also highlighted the links between productive engagement in livelihoods (which is enabled through successful treatment engagement), reduced internalized stigma through restoring people’s perceptions of being productive, active members of the community [36].

There were more novel findings that related to the very rapid ART expansion and the elements of the SEARCH intervention in Kenya and Uganda. The rapid pace of new disclosure events, and changes in care‐seeking behaviour that led many for the first time to access treatment in facilities in their home communities, precipitated the social processes operating on anticipated stigma. We examined evidence for substantial differentiation in the interview narratives across SEARCH study arms, but could not definitively distinguish divergent patterns of change in intervention versus control communities. In short, the declines in stigma and pathways through which this occurred seemed to be present in all study communities; this may reflect the trial’s “active control” design in which large‐scale community mobilization and health campaigns with near‐universal baseline HIV testing in the control communities at the start of the trial accelerated the social changes documented in these qualitative findings.

Prior qualitative research has documented narratives about the power of appearance, or restored health, beauty and vitality in the physical bodies of PLHIV after successful treatment engagement. This phenomenon and its effects on individuals and community norms have been conceptualized in both positive and negative lights: because this leads to PLHIV being able to more easily conceal their status, some researchers have suggested that concealment of status leads to “new forms of stigma.” In some settings studies documented “increased concerns that ART efficacy helps conceal HIV status, allowing PLHIV to intentionally put others at risk through unprotected sex and non‐disclosure [4, 6, 32, 37]. This attitude may change with increasing education and knowledge dissmination regarding the elimination of transmission in PLHIV who are on treatment and achieve viral suppression (i.e. “U = U” messaging). Studies also show reduced disclosure to employers, colleagues and new sexual partners in the context of expanding ART access [38]. We are suggesting here that this stigma is not new: rather, concealment of HIV status is a response to perceived stigma, that is, it is a form of anticipated stigma. We also suggest that the power of appearance, of renewed vitality, may act to circumvent enacted stigma, and consider evidence that this phenomenon may also result in there being fewer triggers of the emotional reaction of others (to signs of illness and decay) that elicit stigmatizing attitudes and manifestations of stigma.

There were counter‐narratives in our data showing that for many PLHIV in the setting, forms of internalized and anticipated stigma continued to exert a negative influence on people’s health‐seeking behaviours and well‐being over the period of observation. Prior literature shows that the ART scale‐up has not been associated with reduced HIV stigma in every setting. It may be that a lack of reduction in stigma contributes to shortfalls to achieving the 90‐90‐90 goals [9], but given that the social processes involved in large‐scale behaviour change are complex, it may also be that testing and ART uptake both influence and are influenced by reductions in stigma. While we present evidence for dramatic changes in stigma, there were also narratives that did not provide evidence of change. It is possible that the time period for observation was too limited to enable ascertainment of large‐scale shifts in social norms, attitudes and practices. We have previously presented findings related to the gendered nature of experiences of stigma as they affect HIV testing [39] and disclosure [40] in the setting; a nuanced presentation of differences across gender and national settings exceeded the scope of the present article. This study is strengthened by its wide scope of inquiry, the large volume of data collected across a heterogeneous cohort of individuals over three years, and the rigor of our approach, which was also enabled through engagement of local researchers in the interpretation of data.

## 5 Conclusions

In conclusion, this study confirms prior research suggesting that declines in HIV‐related stigma may be underway in rural eastern African communities participating in large‐scale UTT implementation. The evidence supports our theoretical model of multiple pathways of stigma decline, showing that declines in stigma may be explained by social processes accelerated by UTT efforts. The implementation of UTT shows promise for reducing stigma via these specific pathways. Such reductions are critical for improving health outcomes among PLHIV, warranting continued investments in the scaling up of population‐level testing and treatment.

## Competing Interests

The authors have no competing interests to report.

## Authors’ Contributions

CSC designed the SEARCH qualitative study, with contributions from EDC, JK, DK and DVH. CSC and MG conducted the analysis; CA, FA, HI, RB, IM, LO, AO and GC contributed to data analysis and interpretation. CSC took primary responsibility and EDC secondary responsibility for writing the manuscript. MG, JK and NS oversaw SEARCH qualitative data collection. DVH, MLP, EAB, MRK and EDC designed and led the SEARCH trial with DK, GC, TDC and CRC and other investigators. All authors were involved in review, critiquing and editing of the manuscript. All authors have read and approved the final version of the manuscript.

## Supporting information


**Data S1.** Follow‐up Community Cohort IDI Guide.

**Data S2.** SEARCH Qualitative Summary sheet‐example.pdf.

**Table S1.** Principal qualitative broad/ “parent” code families for analysis of HIV‐related stigma (community leader and member cohorts), Sustainable East Africa Research in Community Health (SEARCH) study
**Table S2.** Case‐level changes in perceived community norms related to HIV stigma, Sustainable East Africa Research in Community Health (SEARCH) study
