# Supplementary material for: Pathways for reduction of HIV‐related stigma: a model derived from longitudinal qualitative research in Kenya and Uganda
Source: J Int AIDS Soc. 2020 Dec 7;23(12):e25647. doi: 10.1002/jia2.25647 (PMC7720278; doi:10.1002/jia2.25647)
Supplement: Supplementary file 1 — Data S1. Follow‐up Community Cohort IDI Guide. [file JIA2-23-e25647-s001.docx]

**SEARCH Trial Qualitative Cohort**

**SECOND INTERVIEW (Year 1 of SEARCH)**

*[Note to team: prior to interview, prepare Summary Sheets with individualized follow-up questions. Insert the individualized follow up questions into the interview guide below, as needed. Note that questions of clarification for the informant should be asked at the beginning.]*

Thank you again for taking the time to meet with me. Today, I would like to follow up with you on some of the questions we talked about 2 years ago, and then ask you some new questions. *[It would be useful here to remind the informant what you talked about in the last interview. This interview should build on what was described in this last interview. If there are points that need clarification, then those should also be asked in the beginning.]*

COMMUNITY CONTEXT

- In the last interview we had you mentioned the following major problems faced by members of this community *[Recap what the participant told you in the baseline interview here].* Do you still feel these are major problems faced by people in this community?
- Are there any other problems that you feel people now face?
- Have there been any changes since we last spoke, in how people cope with these problems?
- [If the participants do not mention HIV/AIDS as one of the major problems, probe specifically about this.] Is HIV/AIDS a problem in this community?
  - *[If yes:]* How are people in this community coping with the problem of HIV?

COMMUNITY PERCEPTIONS / DISCUSSIONS ABOUT HIV/AIDS

- In the last interview we had you told me that people feel the following way about HIV in this community *[Recap what the participant told you in the baseline interview here].* Do you think people feel the same way today about HIV/AIDS in this community?
- How has the way people feel about HIV/AIDS in this community changed during the past year?
  - Why do you think these changes have occurred?
- Since we last spoke have you known any other people in your community who are infected with HIV?
  - How do you feel about these people?
  - How are these people treated by others in this community?
- Have there been any changes in the way people with HIV are treated by others in this community?
- How has the availability of drugs called antiretrovirals [*or ARVS; also use language informant has previously used, e.g. ‘the big medicine’*] for HIV affected this?
- If someone you know told you that they were infected with HIV, how would you react to them?
- Can you tell me about any experiences you’ve had with people who have disclosed their HIV status to you, since we last spoke?
  - How did you feel at the time?
  - How did you react?
- Have you talked to anyone about HIV/AIDS in your community lately?
  - If yes, can you tell me about one conversation about HIV/AIDS you have had with someone else in this community in the last 6 months. What specifically did you talk about?
  - Why were you talking about this at that time?
- Since the time we last spoke, have you noticed any changes in your community related to attitudes and fears about HIV?
  - What changes have you noticed?
  - How did you notice these changes?
  - Where did you notice these changes?
  - Why do you think these changes occurred?
- Since we last spoke have you noticed any more discussion in your community related to HIV, testing, and treatment?
  - Since the time we last spoke, has your attitude or have your fears about HIV changed?
  - Why do you think these changes occurred?

COMMUNITY PERCEPTIONS / DISCUSSIONS ABOUT HIV TESTING & ARV THERAPY

- When we last spoke you said [*recap what informant said about whether s/he knew people taking ARVs; rephrase follow up question accordingly*]. And now, are you aware of anyone in this community who is taking drugs called antiretrovirals to treat HIV?
  - How did you learn they were taking these drugs?
  - Where do they receive their drugs?
- Do you have any concerns about these drugs that are used to treat HIV? If yes, what are your concerns?
- Since we last spoke have you noticed any more discussion in your community related to antiretroviral therapy for HIV (ARVs)?
- When we last spoke you said [*recap what informant said about whether s/he knew people who had tested for HIV; rephrase follow up question accordingly*]. And now, since we last spoke, have you known anyone in your community who has tested for HIV?
  - *If more people s/he knows have tested for HIV*: What changes have you noticed about how people feel about getting tested for HIV?
  - Why do you think these changes have occurred?
- Where did they test?
- How did you know that they tested?
- Did they tell you the results of their HIV test?
- How did you feel when they told you their test results?
- How did this person feel about their experience with testing?
- *If testing occurred within the community CHC:* Please tell me more about how community members felt about HIV testing at the CHC. How did being able to get tested at the CHC affect this community?
- Do you think that the availability of drugs called antiretrovirals to treat HIV might influence whether a person is willing to test for HIV? *Probe for reasons why / why not.*

PERSONAL EXPERIENCES WITH HIV TESTING, USE OF OTHER SERVICES & DISCLOSURE

- When we last spoke you said [*recap what informant said about his/her previous experience with HIV testing; rephrase follow up question accordingly*]. Have you tested for HIV?
- *If yes*: when did you last test for HIV?
  - Can you tell me more about your testing experience?
  - Why did you decide to test?
  - Did you talk to anyone about your decision to test before you got tested?
  - Where did you test?
    - *If testing occurred at a CHC*: Please tell me more about your experience getting tested at the CHC. How did you feel at the time?
      - How do you think other members of this community felt about getting tested for HIV at the CHC?
      - How did being able to get tested at the CHC affect this community? Please tell me more about that.
    - *If testing occurred at home*: How did it happen that you were tested at home? Please tell me about that.
      - Please tell me about the reasons you decided to get tested at home.
      - [Clarify whether or not home-based testing was provided by SEARCH]
- How did you feel about your testing experience?
  - Did you feel that you got the support you needed during your counseling sessions?
  - Did the counselor answer all of the questions?
  - What would you have changed about the testing experience if you could change one thing?
- How did you feel about learning your HIV test results at that time?
- After you were tested for HIV did you use any specific medical/health services?
  - Tell me about the services you used.
  - Where did you use these services?
  - How did you learn about these services?
  - How did you feel about these services?
  - Are there any services you felt like you needed after you were tested, but you could not access?
- Can you tell me about your experiences sharing your HIV test results with other people after you were last tested.
  - After you were tested, did you talk to anyone about your experience with testing?
  - Have you shared your HIV test results with anyone?
    - *If yes:* whom?
      - Please tell me about your decision to share your test results: Why did you choose this person to disclose to?
      - Please tell me about their reaction when you shared your test results.
      - Were there other people you wanted to disclose your status to, but felt you couldn’t? Please tell me more about that.
    - How has the availability of drugs called antiretrovirals affected your decision about sharing test results with people?
    - *If no*: What makes it difficult for you to share your results?
  - Is there anyone whom you do not want to know your HIV test results?
  - Can you tell me why you do not wish to disclose your test results to this person?
  - What makes it difficult for you to share your results with this person?
  - Did you share your HIV test results with your sexual partner?
    - [*If they mentioned that they had more than one sexual partner last time*]: Did you share your HIV test results with your other sexual partners?
    - Tell me about how you decided which partners to share your results with.
  - How did these partner(s) react when you shared your results with them?
  - How did it feel to discuss testing for HIV with your partner(s)?
  - Can you tell me why you do not wish to disclose your test results to your partner(s) (*or to a particular partner if more than one*)?
    - What makes it difficult for you to share your results with this/these partner(s)?
- *IF THE PARTICIPANT RESPONDS THAT HE OR SHE TESTED FOR HIV PRIOR TO THE YEAR 1 INTERVIEW, AND HAS NOT RE-TESTED SINCE THAT TIME*: You mentioned that you tested for HIV once before the first interview we did together, but you have not been tested again since that time. Can you tell me more about your decision not to test again for HIV?
  - Do you think there is any reason for a person to test more than one time for HIV?
  - If yes, why have you decided not to test again for HIV?
  - Do you think that you may decide to test again at some point? What would be the reasons that would make it likely for you to get tested again?

PERSONAL EXPERIENCES WITH HIV CARE AND TREATMENT [FOR HIV+ INDIVIDUALS]

- Have you ever received any care for HIV?
- *If the participant responds that he or she has NOT previously received care for HIV*: Can you tell me the main reasons why you have not yet received HIV care?
  - Are there any other problems or issues that have kept you from enrolling in care?
  - Please tell me, what would make it easier for you to enroll in HIV care and treatment?

*[FOR PARTICIPANTS WHO HAVE NEVER ENROLLED IN HIV CARE & TREATMENT, SKIP TO NEXT SECTION]*

- *If the participant responds that he or she has previously received care for HIV*: Are you currently receiving regular care?
  - How has receiving HIV care affected your life?
  - Can you tell me please about the drug treatment you are receiving for HIV/AIDS? *Probe whether informant knows what ARV meds are, whether they know what meds they are taking*.
    - *If receiving ARV therapy*: Please tell me about how you learned you were eligible for ARV therapy. Where and when did you learn you were eligible to begin taking ARVs?
      - Who spoke to you about your eligibility for ARV therapy*? Probe for cadre of health care provider and health centre.*
      - How has taking ARVs affected your life?
    - *If not on ARV therapy*: Can you tell me what you have been told about your eligibility or ineligibility for ARV therapy?
      - *If previously told s/he is eligible for ARV therapy, but has not yet started*: Can you please tell about your reasons for not starting ARV therapy?
      - Are there any other reasons you have not started taking ARVs? *Probe for ALL reasons*.
  - Where do you go for medical care? *Probe for all locations*.
  - Have you ever had to change where you access HIV care at any point since you tested positive? *If yes, probe for main reasons why*.
- *If participant not currently enrolled in care:* Can you tell me the main reasons you’re not receiving care currently? *Probe for ALL reasons*.
  - Please tell me, what would make it easier for you to enroll again in HIV care and treatment?
- Can you tell me please, has anyone ever described something called a viral load means?
  - Can you please tell me about your experience learning about your viral load?
  - When and where did you first learn about viral load, and what it means?
  - How has it affected you, to know about your viral load?
  - How important is it to you, to know your viral load?
- Have you ever missed HIV care appointments, or dropped out of care for a time? Please tell me about that.

*[If participant answers no and does not have a problem with missed appointments or dropping out of care, please ask the following question]*

- - Can you please tell about what motivates you to stay in care?
  - What else motivates you?
  - What are the things that help you to keep your appointments?
  - What else helps you keep your appointments?

*[If participant indicates missing care appointments or dropping out of care, please ask the following questions]*

- - What were the circumstances that led this to happen?
  - For about how long was your care interrupted?
  - What are the main barriers you face, to being able to make appointments? *Probe for ALL reasons*.
  - Are there any other reasons?
  - Please tell me, what would make it easier for you to make appointments
- What are the main barriers you face to being able to adhere to taking your HIV medications? *Probe for ALL reasons*.
  - Are there any other reasons?
  - Please tell me, what would make it easier for you to adhere to taking your HIV medications?

RELATIONSHIPS & DISCUSSIONS ABOUT HIV WITH PARTNERS [ALL PARTICIPANTS]

- In the last interview we had together, you told me that you had _______ sexual partner(s) [*Recap what participant mentioned about their sexual partnerships, without referring to a number of partners*]. Are you with the same partner(s) that you were with before?
- Have you had any new sexual partners since we last talked?
- If you are no longer with your former partner(s), can you tell me why your relationships ended?

[*For the following questions, first refer to main partner, then repeat questions for other current/ recent partners*]

- In the last 6 months have you and your partner ever talked about HIV and your risk of HIV?
  - *If yes*: Can you tell me about the conversations you have had with your partner about HIV?
  - *If informant has not talked to partner(s) about HIV or risk of HIV in the last year*: Can you tell me why it is difficult to talk to your partner about HIV?
- *For HIV-negative participants:* Do you feel that you are at risk for HIV because of this partner?
  - *If yes*: Tell me why you think you may be at risk.
    - Do you do anything in your relationship to try to minimize this risk?
- *For HIV-positive participants:* Do you feel any concern about re-infection with a different strain of HIV?
  - *If yes*: Tell me why you think you may be at risk.
    - Do you do anything in your relationship to try to minimize this risk?
- Please tell me about any concerns you feel about infecting your partner with HIV?
  - - Do you do anything in your relationship to try to minimize this risk?
- *ALL participants*: How has the availability of drugs called antiretrovirals to treat HIV affected this?
- Is there anything else you would like to do in your relationship to minimize your risk, yet you feel you are unable to do?
  - *If yes*: Can you tell me why you feel like you are unable to do this with your partner(s).
- Have you talked to your partner about getting tested for HIV in the last year?
  - *If yes*: Can you tell me what you and your partner discussed.
    - How did it feel to discuss testing with your partner?
    - How does your partner feel about getting HIV tested?
  - *If no*: Can you tell me why it is difficult to discuss HIV testing with your partner?
- *For HIV-positive participants*: Have you talked to your partner about accessing HIV care and treatment in the last year?
  - *If yes*: Can you tell me what you and your partner discussed.
  - *If no*: Can you tell me why it is difficult to discuss HIV care and treatment with your partner?

Thank you for sharing your time with me. The information you have given will be very useful for the study.

*Note: Some items in this interview guide were adapted from guides developed by Project Accept (HPTN 043):* Maman S, van Rooyen H, Stankard P, Chingono A, Muravha T, et al. (2014) NIMH Project Accept (HPTN 043): Results from In-Depth Interviews with a Longitudinal Cohort of Community Members. PLoS ONE 9(1): e87091. doi:10.1371/journal.pone.0087091.
