# Supplementary material for: Pathways for reduction of HIV‐related stigma: a model derived from longitudinal qualitative research in Kenya and Uganda
Source: J Int AIDS Soc. 2020 Dec 7;23(12):e25647. doi: 10.1002/jia2.25647 (PMC7720278; doi:10.1002/jia2.25647)
Supplement: Supplementary file 2 — Data S2. SEARCH Qualitative Summary sheet‐example.pdf. [file JIA2-23-e25647-s002.docx]

**SEARCH Qualitative Evaluation:**

**Summary Sheet (Year 1 IDIs)**

**Baseline Interview Summary**

**Transcript file name:**

**IDI Type:** Community Cohort

**Interview Date:** 26-08-14

**Interviewer:** JN

**SEARCH ID:** 20203172062-2

**Gender:** Female

**Other:** Married five year, three children

**Interview Synopsis**

**Community problems/context**

- Water: Bad, salty, far.
- Poverty

**Follow-up:**

**Partnerships**

- Last year you shared with me and you said you had a partner, are you still with him?
- The last time we shared you said your partner had not tested for HIV. How is the situation now?

HIV **testing**

- Last year you said that people say “HIV is the worst disease”. Please tell me what people currently say about HIV.

**HIV Care**

- Last year you said you receiving HIV care from Mbarara and preferred their services as compared to those of Nyamuyanja. Have you changed the location of HIV services? If yes tell me more about that.
- Still when we met, you said you missed some appointment dates. How has this changed over time. You said last year that your CD4 count was high. How is it now?
- You also said that CD4 count was not done at the H/C. Where are you currently having your CD4 cell counts done?

**Health problems**

- The last time we met you said there was no privacy at the H/C. How is the situation now? Last year you also talked about patients being charged ambulance fee. What is the situation now?
- Last year you made a mention of non HIV drug stock outs. Please share with me more on this issue.

**HIV transmission**

- Please tell me more about transactional sex today as compared to how the situation was last year.

**HIV stigma**

- The last time we talked you mention that “people laugh at those who are HIV positive”. How has this changed in the past year?

**HIV disclosure**

- From the time we talked are there people you have disclosed to other than your family members?
- Poor roads
- Distant health facility

**Community health problems**

- Distant health facility
- No CD4 count done at H/C
- Ambulance fee of 40000=
- Shortage of non HIV drugs

**HIV Services**

Poor HIV services in terms of;

- No CD4 done at H/C
- No available treatment for other illnesses outside HIV
- Client has been on ART for three years and prefers to receive HIV care from Mbarara hospital.
- Missed appointments.
- Lack of privacy when accessing care.

**HIV Testing**

- Encouraged by her aunt to go and test for HIV
- Reason for the test was due to the x boyfriends death and the sore throat she had
- Preferred to test from Mbarara hospital
- Partner refused to Test for HIV

**HIV test results disclosure**

- Disclosed to partner and close relatives and friends
- Community members fear to disclose to their marriages.

**Reasons for high HIV prevalence in the** **community**

- Transactional sex
- Lack of condom use

**Attitudes towards HIV**

- Stigma (“people are laughed at”, “people do not love themselves”)
- People say “HIV is the worst disease”

**ART/ARVs**

- Experienced no ART side effects however people reported; headache, dizziness.
- ARV availability has encouraged people to test for HIV.
- Never missed medication.
- ARVs help to elevate CD4 and people look good.

**SEARCH concerns**

People associate SEARCH to HIV

**Partnerships**

- Has had previous multiple sexual relationships

*Note: Summary sheet adapted from an instrument developed by Project Accept (HPTN 043):* Maman S, van Rooyen H, Stankard P, Chingono A, Muravha T, et al. (2014) NIMH Project Accept (HPTN 043): Results from In-Depth Interviews with a Longitudinal Cohort of Community Members. PLoS ONE 9(1): e87091. doi:10.1371/journal.pone.0087091.
