# Supplementary material for: Pathways for reduction of HIV‐related stigma: a model derived from longitudinal qualitative research in Kenya and Uganda
Source: J Int AIDS Soc. 2020 Dec 7;23(12):e25647. doi: 10.1002/jia2.25647 (PMC7720278; doi:10.1002/jia2.25647)
Supplement: Supplementary file 3 — Table S1. Principal qualitative broad/ “parent” code families for analysis of HIV‐related stigma (community leader and member cohorts), Sustainable East Africa Research in Community Health (SEARCH) study Table S2. Case‐level changes in perceived community norms related to HIV stigma, Sustainable East Africa Research in Community Health (SEARCH) study [file JIA2-23-e25647-s003.docx]

**Table S1: Principal qualitative broad / “parent” code families for analysis of HIV-related stigma (community leader and member cohorts), Sustainable East Africa Research in Community Health (SEARCH) study**

| **HIV stigma** | Discussions of HIV-related stigma and discrimination, including perceived stigma in the family/household or community, internalized stigma (e.g. shame, low self-worth due to HIV), and enacted stigma (e.g. social exclusion, discrimination, violence). Discussions about consequences of being HIV-positive related to stigma, such as loss of income/livelihood, loss of marriage and childbearing options, poor care, loss of hope or feeling of worthlessness, and loss of reputation. |
| --- | --- |
| **Cultural practices** | Specific cultural practices mentioned by community members (e.g. widow inheritance, polygamy, other community-specific cultural practices) |
| **HIV knowledge** | Description of how the respondent became aware of HIV and general knowledge about HIV/AIDS in the community. Includes means through which an individual or community members become aware about issues concerning HIV in general. |
| **HIV perceptions** | Description of respondent or community attitudes and perceptions about HIV/AIDS in the community, or descriptions of community attitudes and perceptions about HIV/AIDS in the community. |
| **HIV severity** | Any discussion about how big of a problem HIV/AIDS is in the community: whether HIV is a bigger or smaller problem than other issues in the community, the severity of HIV/AIDS in the community currently, HIV severity now as compared to the past. |
| **HIV transmission** | This includes participant’s or community’s opinions/views on how HIV is being spread in their community; description of who or what they think is contributing to HIV transmission in their community. |
| **HIV prevention** | The measures/ways in which people have tried to avoid/prevent themselves from becoming infected with HIV. Methods of reducing or eliminating the risks of HIV transmission (e.g. use of condoms, reducing numbers of partners / being faithful to one's partner, abstinence from sexual activity; also includes discussion of ARVs and undetectable viral loads of known HIV+ partner / treatment as prevention.) |
| **PLHIV-personal** | Discussions of personal perceptions (positive or negative) about how participant views PLHIV: does the participant know someone living with HIV, have they known someone who has died of HIV/AIDS, how do they feel about PLHIV, how do/would they react to someone telling them they had HIV, beliefs about PLHIV. |
| **PLHIV-community** | Discussions of community norms/attitudes/beliefs regarding PLHIV (can be positive or negative). |
| **HIV advocacy** | Description of personal experiences with speaking to people about HIV/AIDS generally, giving advice or counseling people in the community/friends/relatives. |
| **HIV conversations** | The description of the last specific conversation the participant had regarding HIV/AIDS. |
| **HIV testing- personal** | Discussion of personal experience with testing, personal motivation to test for HIV. Includes discussions about knowledge of HIV status: avoiding knowing status, wanting to know status, feelings and beliefs about knowing one’s HIV status. |
| **HIV testing- community** | Perceptions of community norms regarding HIV testing in the community. Includes discussions about how others feel/think about knowledge of HIV status: avoiding knowing status, wanting to know status, feelings and beliefs about knowing one’s HIV status. |
| **HIV disclosure- personal** | Description of whether the participant has personally disclosed his/her status/test results to anyone, how they chose that person to whom to disclose, what made him/her decide to share his/her status/test results, whether there are others to whom they would like to disclose but feel they cannot, what inhibits disclosure, means through which disclosure is made easier. |
| **HIV disclosure-community** | Discussion of norms around disclosing one’s status/test results with others, motivation to do so or barriers not to do so. |
| **HIV care experiences** | Description of whether or not the participant, if HIV-positive, is currently receiving regular HIV care including how HIV care/treatment has affected a participant’s life, where they go for treatment, why they decided to go to that particular site, whether access to treatment has changed over time. |
| **HIV care barriers** | Discussion on PLHIV by a participant about missing an HIV care appointment, dropping out of care for some time, barriers to care including negative experiences with providers, issues of stigma and discloser, problems with money or transportation, depression, etc. Also, what would make it easier to make appointments. |
| **Partnerships** | Participant describes sexual partnerships, marriage relationship, and any current sexual history. Also, whether the participant has ever considered his/her HIV risk in relation to that partnership or tested as a couple. |
| **HIV serodifferent partnerships** | Participants describing being in any known serodifferent relationships, whether with primary or other partners. |

Abbreviations: People living with HIV (PLHIV)

**Table S2: Case-level changes in perceived community norms related to HIV stigma, Sustainable East Africa Research in Community Health (SEARCH) study**

| **Cohort member** | **Baseline year** | **Follow-up** |
| --- | --- | --- |
| Male community leader, eastern Uganda | [I: *Why do some people fear to disclose their HIV status to those whom they are close to, their partners, children or others*?] Some people fear to be segregated… I can also talk about fear of missing opportunities in the future if people learnt that you are HIV-positive... like political and leadership opportunities […] | *Year 2:* [I: y*ou told me last year that people segregate those with HIV and they couldn’t easily give them responsibilities like leadership, thinking that they that they are going to die soon. Do people still hold the same feeling?*] No, the opposite is true. Nowadays they know that even though someone is HIV positive, he can be productive, bear children and run his or her daily activities normally, even leadership… Previously the feeling was that as long as you are infected with HIV, the only thing you have to wait for is death. Today this has changed. People can now live positively with HIV. Partly that is because of the SEARCH activities which caused a lot of awareness about HIV in the community. |
| Female community member, HIV-positive, western Kenya | [I: *How do people feel about HIV/AIDS in this community*?] Many live in fear… that others might make their status public. [I: *Is that a common practice in this community*?] Yes, it is still there. We are treated normally but many tend to keep off from those at AIDS stage… I wonder why, yet HIV does not walk to another person…(*laughter)*. | *Year 2:* People are just living well with one another, not like before when an infected person could get a negative remark from others… such painful remarks that would make one to even drop care. These days you hear people saying “*HIV is good… comparing it with cancer, cancer is bad, when you get infected you just die, but HIV, once I have taken my drugs then I just do my activities like any other person*”. People have now come to accept HIV like any other disease and they don’t fear it anymore. |
| Female community member, HIV-positive, western Kenya | They used to say “*You cannot cook porridge in this school if you are not HIV positive”.* …All the community members were aware [*that people who participated in a program at a local school were HIV-positive*] and it created discrimination against the infected. We were looked down upon... | *Year 2:* At first, many people boycotted [*HIV testing campaigns*]. Those who agreed to be tested became good ambassadors in the community and really motivated others to test and that is where the change started… people urge each other to test for HIV. People used to fear very much those who are HIV positive and could not even share a meal with them, but all these are long gone because people have known the truth. There is equal treatment of any human being in this community. |
| Male community member, HIV-positive, western Kenya | [*People living with HIV*] fear […] their HIV status being disclosed publicly without the victim’s consent. It is still in people’s mind that HIV kills, and not a condition that one can manage to prolong his or her life. This is why some people shy away from disclosing to their children. | *Year 2*: I can say there are some changes, nowadays when an individual has tested HIV positive, they easily embrace the HIV treatment […] This is because we have people whose health had greatly deteriorated, and once they started using the drugs, their lives changed and they are living healthily and performing their chores as usual. [*Interviewer: How are HIV-positive people treated by others in this community*?] […] It is better nowadays in the way they are treated, compared to before.  *Year 3*: In my area, most people are living with HIV and so you cannot look down on another person because we are on the same boat [...] [*Interviewer: What about the insults that were there about those who have HIV?* ] That is currently going down. […] This is because in my area almost everyone is infected. […] In the past, people would have bad coughs, diarrhea, weight-loss, but now these are not there. You may also find that when you go to the hospital for the drugs, you meet someone who looks completely healthy and you can’t imagine they are infected. This makes you realize that you are not alone in this. |
| Male community member, HIV-negative, western Kenya | [*People living with HIV*] ought to have been isolated from the non HIV-infected persons […]  We do say that these drugs [*antiretroviral therapy*] will subject the community into more deaths, as those using them appear as healthy as others. These “healthy” individuals also have a tendency of looking for sexual partners regardless of their status, which leads to more infections in the community. | *Year 2*: Right now they [*People living with HIV*] are treated very normal… when I spoke with you last they were still being treated with much disdain. […] Because the people that were known to be HIV positive were few… right now it is very common. […] There is a friend of mine who told me that he was going to the clinic and at first I could not understand what he meant… when he came back he disclosed to me that he was HIV positive. […] I was really touched by his revelation. He thought that after disclosing to me his status I would then tell others his HIV status but he has never heard of it from any other person indicating that I kept silent with what he told me. So every time he is faced with any difficulty he comes and confides in me…  *Year 3*: In my own opinion and personal research, have come to realize that each and every home we will realize that one or two people are infected or affected in one way or the other. They are also our family members hence we can’t ignore them. |
